# Comparison of the Status of Sleep Quality in Basic and Clinical Medical Students

**DOI:** 10.7759/cureus.4326

**Published:** 2019-03-26

**Authors:** Munazza Khero, Maham Fatima, Mir Ali Asghar Shah, Amber Tahir

**Affiliations:** 1 Internal Medicine, Jinnah Sindh Medical University, Karachi, PAK; 2 Internal Medicine, Jinnah Postgraduate Medical Center, Karachi, PAK; 3 Internal Medicine, Dow University of Health Sciences, Karachi, PAK

**Keywords:** sleep quality, impaired sleep, sleep disturbances, pittsburgh sleep quality index, medical student

## Abstract

Introduction

Academic pressure and its associated stress are responsible for disturbances in the circadian cycle of the students. Adequate sleep has crucial role in enhancing cognitive skills especially memory retention. Poor night time sleep quality and consequent daytime sleepiness affects physical and cognitive health of the students. In this study, sleep quality among medical students is evaluated.

Methods

It was an observational, cross-sectional study conducted with undergraduate medical students. Pittsburgh Sleep Quality Index (PSQI) was used to assess the sleep quality. The data was analyzed using Statistical Package for the Social Sciences (SPSS) 22.0 (IBM Corp., Armonk, NY, USA).

Results

Of 281 students, 155 (55.16%) were pre-clinical students and 126 (44.83%) were clinical students; 207 (73.66%) were female students and 74 (26.33%) were male. The overall frequency of poor sleepers was 172 (61.2%); 95 (55.2%) of these were clinical sciences and 77 (44.8%) were basic sciences students. Sleep latency, duration, and efficiency were more impaired in clinical students (p-value ≤ 0.05). Use of sleep medications and daytime dysfunction was more common in clinical students (p-value ≤ 0.05).

Conclusion

Medical students are continuously under high academic stress and pressure. Adequate sleep is essential for them to refresh them every day and help in learning and memory processing. Medical students in advance years of education have worse sleep quality than those in early years. Efforts should be taken to improve their sleep quality in order to protect the psychological and emotional health of future doctors.

## Introduction

Academic pressure and its associated stress are responsible for disturbances in the circadian cycle of the students [1]. Unhealthy lifestyle habits such as overuse of internet and television especially at the night time [2], and smoking further deteriorate sleep quality [3]. Research has repeatedly given evidence that medical students have more severe sleep impairment than students of other fields [4]. The factors responsible include longer study duration, harder syllabi, exam stress, clinical duties which include overnight on-call duties, and emotional challenges and exhaustion associated with witnessing human misery [5, 6].

Adequate sleep has crucial role in enhancing cognitive skills especially memory retention. Poor night time sleep quality and consequent daytime sleepiness affects physical and cognitive health of the students. It deteriorates everyday performance of these students [7] and has negative effects on their emotional health including empathy [8].

Various studies conducted all across the world including Iran [9, 10], United States of America [11], Brazil [12], and Lithuanian [13] have consistently shown poor sleep quality among medical students. A study comparing the sleep quality of medical students and non-medical students suggested that medical students had higher incidence of poor sleep quality and associated poor quality of life as compared to students in other fields of education [13]. In a study conducted in 2015 among medical students of Karachi, it was concluded that almost 40% of medical students are classified as poor sleeper [14].

Medical students have a stressful academic career, so it is important to identify those students with sleep issues, extent of issues and factors contributing to it. In this study, sleep quality among medical students in Karachi is evaluated.

## Materials and methods

It was an observational, cross-sectional study conducted with undergraduate medical students in Jinnah Sindh Medical University, Karachi. The study duration was from 15th September till 15th November 2018. Students of both genders, and all five years of education were included. With an estimated prevalence of 66% [15], confidence interval of 95% and absolute precision of 0.05, the sample size for this study was calculated to be 281. Written informed consent was taken from all students. The study was approved by the institutional review board.

A structured questionnaire was made. It included two parts. The first part comprised of demographic information including gender, age, and year of education. Year of education was divided into basic sciences students - first and second year - and clinical sciences students - third, fourth, and final year students. The second part comprised of Pittsburgh Sleep Quality Index (PSQI). PSQI is an efficient measure of the quality and pattern of sleep. It assesses sleep quality on seven components - subjective sleep quality, sleep latency, sleep duration, habitual sleep efficiency, sleep disturbances, use of sleeping medication, and daytime dysfunction. Its score ranges from minimum zero to maximum twenty-one. The combined score of all seven components is termed as “global score of PSQI.” Global PSQI score ≥5 signifies “poor sleep quality.” The PSQI has internal consistency and a reliability coefficient (Cronbach's alpha) of .83 for its seven components [16].

Statistical Package for the Social Sciences (SPSS) for Windows, version 22.0 (IBM Corp., Armonk, NY, USA) was used to enter and analyze the data. Demographic data was categorized to calculate frequencies and percentages. All components of PSQI were categorized as mentioned in Smyth [16]. All scores of PSQI were compared for basic sciences and clinical sciences students. Chi square was applied for calculating statistical significance. P-value of ≤0.05 was taken as significant.

## Results

Of 281, 155 (55.16%) participants were pre-clinical students and 126 (44.83%) were clinical students. There were 207 (73.66%) female participants and 74 (26.33%) male participants. From the entire sample, the mean PSQI score was 39.52 + 11.87. There were 172 (61.2%) students who scored ≥5 on global PSQI indicating poor sleep quality. There were 95 (55.2%) clinical sciences students with poor sleep quality and 77 (44.8%) basic sciences students. On all seven components of PSQI, the sleep quality between basic sciences and clinical students was compared. Fairly bad to very bad subjective sleep quality was seen in 58.7% basic sciences students and 66.7% clinical students. Sleep latency, duration, and efficiency were more impaired in clinical students (p-value ≤ 0.05). Use of sleep medications and daytime dysfunction was more common in clinical students (p-value ≤ 0.05) as seen in Table 1.

**Table 1:** Comparison of Pittsburgh Sleep Quality Index (PSQI) Scores on seven components in basic and clinical medical students.

Components of PSQI	Basic sciences students (n = 155; 55.2%)	Clinical students (n = 126; 44.8%)	p-value
Subjective sleep quality			
Very good	28 (18.0%)	18 (14.2%)	0.50
Fairly good	36 (23.2%)	24 (19.0%)	
Fairly bad	43 (27.7%)	44 (35.0%)	
Very bad	48 (31.0%)	40 (31.7%)	
Sleep latency			
≤15 min	28 (18.0%)	23 (18.2%)	0.01
16-30 min	52 (33.5%)	26 (20.6%)	
31-60 min	43 (27.7%)	56 (44.4%)	
>60 min	32 (20.6%)	21 (16.6%)	
Sleep duration			
>7 h	61 (39.3%)	27 (21.4%)	0.007
6-7 h	25 (16.1%)	32 (25.4%)	
5-6 h	35 (22.5%)	39 (31.0%)	
<5 h	34 (21.9%)	28 (22.2%)	
Habitual sleep efficiency			
>85%	32 (20.6%)	22 (17.4%)	0.03
75%-84%	67 (43.2%)	43 (34.1%)	
65%-74%	38 (24.5%)	30 (23.8%)	
<65%	18 (11.6%)	31 (24.6%)	
Sleep disturbances			
0	38 (24.5%)	26 (20.6%)	0.56
1-9	40 (25.8%)	27 (21.4%)	
10-18	38 (24.5%)	38 (30.1%)	
19-27	39 (25.2%)	35 (27.7%)	
Use of sleep medication			
Not during the past month	85 (54.8%)	48 (38.1%)	<0.00001
Less than once a week	47 (30.3%)	29 (23.0%)	
Once or twice a week	19 (12.2%)	35 (27.7%)	
Three or more times a week	4 (2.5%)	14 (11.1%)	
Daytime dysfunction			
1-2 days	73 (47.1%)	28 (22.2%)	0.0001
3-4 days	42 (27.1%)	42 (33.3%)	
5-6 days	24 (15.5%)	30 (23.8%)	
Everyday	16 (10.3%)	26 (20.6%)	

## Discussion

The study suggests poor quality of sleep among medical students of Karachi. Clinical sciences students are more gravely affected by sleep disturbances as compared to basic sciences students. Clinical sciences students have shown significantly lower sleep latency, duration, and efficiency, with more frequent sleep disturbances and daytime dysfunction. Use of sleep medications was also more common in clinical sciences students. However, subjective sleep quality was comparable in both groups.

Studying medicine at undergraduate level is highly-demanding. Physical and psychological well-being of medical students is essential for learning, adaptation, and mastering their roles as future doctors. Their own health and their attitudes towards healthy lifestyles will substantially influence their future practice [6]. Learning and memory processing is greatly influenced by adequate sleep. In students, sleep-impaired sleep quality can damage academic performance [4] and cause emotional exhaustion and burnout. Burnout, in return, further aggravates sleep disturbances among medical students [5]. Among Iranian medical students, 40% are poor sleepers; most of which are medical interns. Poor sleep quality is associated with bad grades in their students [9]. They reported 24% of poor sleepers to be basic sciences students in comparison to 45% in this study [9].

In a comparative analysis with law, economic, and medical students, the worst sleep quality was reported in medical students. In comparison to students of other majors, medical students spent more hours studying, were more stressed about their studies and less satisfied with results, and more often studied just before going to bed [13]. Older studies from Pakistan have shown 40% poor sleepers with impaired sleep quality more common in female students. However, association with year of education could not be established [14]. However, in a study with Brazilian medical students, 40% students reported bad sleep quality with sleep disturbances to be more common among the students of first and second year of medical school. They attributed their results to inadequate sleep hygiene among young students including poor social life and late-night eating and internet surfing habits [17]. In contrast, in this study older students reported more frequent sleep impairment. This might be because of the increased academic pressure in higher years of education and the stress of managing theoretical studies and clinics together. However, further studies are needed to reinforce this relationship.

The study has its limitations too. It was only done in one institute and cannot be generalized. It did not take into consideration reasons predisposing to impaired sleep and also did not take into account the consequences of disturbed sleep. We propose prospective studies to assess the consequences of sleep deprivation on the psychological and physical health of these students. We also propose controlled trials to evaluate whether adequate sleep actually improves the psycho-social health and academic performance among medical students.

## Conclusions

Medical students are continuously under high academic stress and pressure. Adequate sleep is essential for them to refresh them everyday and help in learning and memory processing. Sleep disturbances are common in medical students and worsen as the years in education advance. Efforts should be taken to improve their sleep quality in order to protect the psychological and emotional health of future doctors.
